# Understanding and Preventing Health Concerns About Emerging Mobile Health Technologies

**DOI:** 10.2196/14375

**Published:** 2020-05-25

**Authors:** Frank T Materia, Kate Faasse, Joshua M Smyth

**Affiliations:** 1 The Pennsylvania State University University Park, PA United States; 2 University of New South Wales Sydney Australia

**Keywords:** mHealth, technology, nocebo effect, implementation science, medically unexplained symptoms

## Abstract

New technologies and innovations have often improved population well-being and societal function; however, these are also often initially accompanied by worry and fear. In some cases, such worries can impede, or even prevent entirely, the adoption of the technology. Mobile health (mHealth), a discipline broadly focused on employing ambulatory technologies to improve the affordability, reach, and effectiveness of health promotion and clinical intervention approaches, offers new innovations and opportunities. Despite emerging evidence supporting mHealth efficacy (eg, for improving health outcomes), some individuals have concerns about mHealth technology that may impede scalability, efficacy, and, ultimately, the public health benefits of mHealth. We present a review and conceptual framework to examine these issues, focusing on three overarching themes: biophysiological, psychological, and societal concerns. There are features of mHealth that lead to worries about the potential negative effects on an individual’s health (eg, due to exposure to electromagnetic or radio waves), despite evidence supporting the safety of these technologies. When present, such beliefs can lead to worry that gives rise to the experience of unpleasant and concerning physical symptoms—the nocebo effect. This may represent an important implementational barrier because of apprehension toward beneficial mHealth products (or features thereof, such as wireless charging, wearable or implantable sensors, etc) and may also have broader ramifications (eg, leading to economic, governmental, and legislative actions). In addition to reviewing evidence on these points, we provide a broad three-step model of implementation research in mHealth that focuses on understanding and preventing health concerns to facilitate the safe and effective scalability of mHealth (and that may be generalizable and applied to similar technologies): (1) evaluating and better discerning public perceptions and misperceptions (and how these may differ between populations), (2) developing theory-based public health communication strategies regarding the safety of mHealth, and (3) disseminating this messaging using evidence-based methods. Collectively, these steps converge on reviewing evidence regarding the potential role of worry and nocebo in mHealth and providing a model for understanding and changing attitudes and preventing unfounded negative perceptions related to mHealth technology.

## Introduction

Human technological innovation has progressed tremendously over the past century. From the flagship electronic devices of the early 1900s to the advanced circuits of the midcentury, the proliferation of the personal computer and the internet in the 1980s and 1990s, and the current expansion of nanotechnology and smart devices, the rapid evolution of technology presents opportunities for improving societal function and global well-being. One particular scientific field, *mobile health* (mHealth), is an example of technological expansion aimed at improving human outcomes.

Over the past 20 years, mHealth has emerged as an integrative discipline, focusing on developing and implementing wireless, portable, or implantable technology for improving human health [1-3]. Some mHealth approaches such as wearable fitness trackers for encouraging physical activity and smartphone apps supporting medication adherence have become ubiquitous in modern society as tools for promoting health [4]. Infrastructures across the world, at multiple levels, continue to adapt to support the introduction of mHealth devices to the masses. Some examples include investments in expanding cellular infrastructure and internet access [5], advertising campaigns disseminating new mHealth products [6], collaborations between engineers and physicians for developing innovative mobile intervention approaches [7], and electronic systems integrating mobile technology into clinical treatment approaches within health care organizations [8,9]

## Mobile Health Technology’s Potential for Improving Health

The rapid proliferation of new technologies has often outpaced the slower process of collecting evidence on mHealth, such as by conducting rigorous clinical trials [1]. However, there is a growing scientific evidence base supporting mHealth’s effectiveness in improving health outcomes. Recently published clinical trial results, accompanied by evidence derived from alternative research methodologies (eg, microrandomized trials and *big-data* analytic approaches), increasingly support the efficacy [2] and safety [10] of mHealth approaches for improving various health behaviors and outcomes (eg, smoking cessation [11], HIV care [12], medication adherence [13], chronic disease management and care [2], and health-related quality of life [14]) and reducing traditional care costs [15].

## Need for Human-Centered Implementation Research

Even with emerging evidence suggesting the benefits of mHealth, the expansion of such technologies across multiple levels of society worldwide calls for *implementation* research—investigations aimed at better understanding the factors related to the successful introduction and utilization of mHealth interventions in the real-world community and clinical settings [16]. Although limited, some mHealth implementation research does exist, and there are frameworks developed (eg, the Integrate, Design, Assess, and Share – IDEAS Framework [17]) for digital interventions that broadly suggest methods for designing, implementing, and disseminating such products. Overall, however, the literature regarding best practices for implementing mHealth is unclear. There is some consensus that one of the most crucial prescriptions for advancing mHealth implementation research involves better understanding the human-centered factors associated with the adoption, uptake, and sustained use of new technologies [18]. Even if mHealth systems are shown to be effective, if individuals are unwilling to accept, trust, and engage with such technologies, mHealth cannot reach its potential for improving public health.

## Issues in Widespread Technology Adoption

Given there is no *gold standard* for conducting human-centered mHealth implementation science [19], historical perspectives regarding mass technology introductions may provide an evidence-informed precedent for approaching such research questions. Large-scale technological implementations of the past century provide exemplar accounts of human-centered factors that may hinder the widespread implementation and utilization of mHealth. For example, unsupported health worries surrounding the safety of microwave ovens (ie, concerns about microwave radiation being emitted into the nearby environment, increasing a perceived risk of cancer) date back to the product’s introduction in the 1940s [20], and these worries were further exacerbated in the 1960s when a US government survey reinforced this (mis)perception [21]. The expansion of mobile phones and cellular networks in the 1980s brought about public trust issues with the increased amount of unseen radio waves traveling through the air [22]. Recently, public concerns have been raised about the use of wind turbines for sustainable energy and almost unavoidable exposure to Wi-Fi internet signals and related to the upcoming global transition to 5G cellular data connectivity [23-27]. These factors tend to revolve around worries and distrust about the effects that unfamiliar, innovative devices may have on physical well-being [28]. Although many people support such advancements, some are hesitant and express concerns about potential risks—particularly about negative health effects—of new technologies [29].

In addition, the recognition that large corporate entities (eg, tech companies, banks, and communication providers) increasingly capture large amounts of data for their own use—often without clear disclosure and, in some cases, without consent—has stoked concerns about data privacy and the usage of such data [30,31]. These data concerns, coupled with existent worries about the risks of new technologies on physical health, have led to distrust and may ultimately culminate in technological avoidance behaviors [32].

## Biopsychosocial Approach to Understanding Mobile Health Concerns

We propose that worries about new technologies are multifaceted and that implementational concerns can be characterized as stemming from the dynamic interplay of biophysiological (eg, concerns about mHealth affecting physical health), psychobehavioral (eg, affecting well-being, perceptions, and decision making), and social (eg, affecting larger global policy and regulatory practices) constructs. Given mHealth’s integration and reliance upon varied technological platforms (eg, passive sensing, wearable components, near-field communication [NFC] transmitters), we view mHealth technology as a case study of how current widespread implementation of innovative devices (with great potential for improving human health) may inadvertently instigate public distrust and concern. These issues likely are not confined to mHealth; many related technological services and products (eg, wireless power transfer, smart homes, and Wi-Fi connectivity) serve other market interests beyond health promotion and intervention yet may share features that similarly elicit concerns or distrust. We thus hope our attempt to better understand and prevent biopsychosocial public concerns with mHealth may help inform related technologies and issues.

## Objectives

The overarching objective of this paper is to bring attention to the potential biophysiological, psychological/behavioral, and societal ramifications related to (often unfounded) health concerns with newly emergent mHealth technologies. Given that there is very limited existing literature about this phenomenon, it is not yet possible to conduct a systematic review on this topic. Rather, we aimed to narratively summarize the key issues, present selected evidence, and, more generally, provide a conceptual framework for these issues—particularly for readers who develop novel mHealth technology (including those outside of social and behavioral science, such as engineers). Notably, we posit that there already likely are—and will continue to be—potential implementation barriers in the rapidly emergent mHealth product market. Such barriers, if not evaluated and addressed, may lead to lower uptake and nonadherence with mHealth technology. Specifically, we highlight the potential role of concerns regarding *power* (eg, electromagnetic hypersensitivity syndrome, EHS) and the nocebo effect, consumer distrust and technology avoidance behaviors, and larger social implications for user acceptability, regulation, and market availability. We also attempted to indicate how such considerations may extend to other (similar) technologies besides mHealth.

The absence of empirical evidence in this context also means the prevalence and magnitude of mHealth worries and concern are, at this point, somewhat left to speculation. To address this dearth in the literature, we believe it is essential to outline a conceptual framework to stimulate and inform needed research. Therefore, a secondary goal is to propose an approach to studying the details of such mHealth-related worries and concerns and suggest public health prevention efforts for curbing unfounded distrust in mHealth technologies. The proposed framework also integrates elements from widely accepted models for implementation research (eg, the Reach, Effectiveness, Adoption, Implementation and Maintenance-RE-AIM Framework [33] and the Consolidated Framework for Implementation Research – CFIR [34])—broadly speaking, evaluating the target population’s perceptions, designing interventions to match needs, and disseminating materials with evidence-informed methodologies. Future directions for evaluating and enhancing the uptake of safe and effective mHealth technologies are presented.

## Biophysiological Concerns With Mobile Health Implementation

Worries about how novel technologies influence health outcomes are prevalent and appear to be increasing with the proliferation of advanced modern technology [29]. Many new technologies, just as have many *old* technologies over the past century, have been linked to public concerns and reports of unpleasant physical symptoms and adverse health outcomes that sufferers attribute to exposure to new technologies [35]. Symptoms reported are typically not associated with a specific bodily system or an underlying disease process but, nonetheless, are reported as severe and disabling; these include, for example, sleep disturbances, fatigue, headaches, and cognitive problems [35]. Such concerns about the potential harms of new technologies and other aspects of modern life are common among otherwise healthy individuals [36]. Public concerns appear to be heightened by technological exposures that have certain characteristics, for example, exposures that are involuntary, inescapable, and from a novel or unfamiliar source [37].

### Electromagnetic Hypersensitivity Syndrome and Mobile Health

One feature common to many technologies, including many mHealth devices, that prompts public concerns about health impacts is the emission of electromagnetic radiation. A number of products and components necessary for supporting mHealth systems (eg, Wi-Fi routers, cellular towers, rechargeable batteries, and wireless data) rely on power to operate harness magnets, capture or emit radio waves, or send or receive electricity. Some individuals have concerns about the release of unsafe levels of electromagnetic radiation into the environment. Furthermore, it is purported that there are specific types of predisposed or particularly sensitive individuals who experience unpleasant symptoms following such perceived exposures. Such people are often described as having EHS [35]. Some studies have estimated that around 1 in 20 people in the general population believe they are affected by EHS, and it appears that many more are concerned about the potential negative health effects of exposure to electromagnetic radiation [38,39].

It is likely that media coverage can strongly shape individuals’ perceptions toward potentially inaccurate (and sometimes risky) misinformation (eg, the antivaccination movement [40] and dangerous diet fads [41]). In recent years, the reporting of EHS in the media has increased public concerns and subsequently reported instances and symptoms, as attributed to electromagnetic frequencies in the environment [42]. Such circumstances have led to general public distress and distrust, increased seeking of medical care for EHS symptoms, and a growing number of activists and community groups worldwide [24]. Some EHS sufferers and their families have filed lawsuits to get rid of technology that they view as posing risks to environment (including those vital to mHealth, such as Wi-Fi), asserting that such exposures are the cause of negative physical and psychological health outcomes, including suicidal ideation [43]. Other EHS sufferers have taken more extreme intervention measures, such as choosing to leave their families and re-establish their lives in desolate rural areas where cellular networks are nonexistent [24]. We recognize that the veracity of claims from media outlets can be questionable; what these reports do suggest, however, is that concerns and worries related to technology appear prominent, broadly distributed (eg, geographically and demographically), and accompanied by behavioral responses intended to manage the concerns and worries.

### Science Behind Electromagnetic Hypersensitivity Syndrome

In response to rapidly growing public concerns, observational and experimental studies have been conducted to understand the prevalence and possible physiological mechanisms of EHS. Cross-sectional research has found that the majority of EHS sufferers are self-diagnosed and have not sought medical care for the condition, that the mean duration of EHS symptoms is 10.5 years, and that more than half of those with EHS actively try to avoid electromagnetic field (EMF) sources [44]. Rigorous trials (both in the lab and out in the field) over the past 20 years have also been conducted to better understand EHS and the effects of electromagnetic radiation on human health. The general consensus of the scientific and medical communities, based on extensive evidence, including that from double-blind sham-controlled experimental studies, is that the symptoms reported by individuals with EHS are *not* caused by exposure to electromagnetic radiation. When people believe that they are (or might be) exposed to EMF, they report experiencing unpleasant EHS symptoms, regardless of whether the EMF-emitting devices are switched on or off [35,45-48]. Multiple systematic reviews have found that there is no evidence to support EHS as a disease with a biological basis [49,50] and that there has never been a causal relationship established between exposures to electromagnetic radiation and well-being [51]. An alternative viewpoint that EHS symptoms are likely to be caused by the expectations and beliefs about the risk of potential harm and the accompanying worry is emerging; this process has more broadly been described as the nocebo effect.

### Nocebo Effect

This is a phenomenon whereby nonspecific, unpleasant physical symptoms and other adverse health outcomes occur in response to negative expectations and beliefs (in this case, related to exposure to electromagnetic radiation)—rather than any specific physiological or environmental cause [28,52-54]. Nocebo effects are prevalent in medicine and daily life [55], including in the development and maintenance of EHS symptoms, and these effects can be powerful enough to cause symptoms that lead individuals to hospitalization and medical intervention [56]. Although the empirical evidence shows that exposure to multiple types of invisible wave frequencies does not cause EHS, individuals are still reporting unpleasant symptoms and negative outcomes as a result of (real or perceived) exposure. As noted, the (likely mistaken) perception that EMF exposure is unsafe leads to experiencing associated concerns, negative expectations, and subsequent (nocebo) symptoms.

Negative expectations can act via two different pathways to cause nocebo effects. First, by directly influencing the experience of physical symptoms through neurobiological changes as well as increasing anxiety and shifting attention toward the expected experience [57]. Second, via a misattribution process whereby normal physical symptoms are mistakenly attributed to the perceived toxic or harmful exposure (ie, in this case, electromagnetic radiation). The experience of physical symptoms is common, even in healthy individuals, thereby providing a range of potential symptoms for misattribution at any given time [58].

### Mobile Health Innovations May Fuel Nocebo Effects

Given the possibility that negative expectations regarding the perceived risk resulting from exposure to electromagnetic radiation can fuel nocebo symptoms and the common use of EHS-relevant technologies in the mHealth field (eg, Wi-Fi, wireless charging, Bluetooth connectivity, cellular signals, etc), evaluating and addressing worries about mHealth technologies is timely. Technologies that are poorly understood by the population at large are increasingly being employed to support mHealth prevention and treatment approaches. For example, wireless power transfer is now necessary for charging current health-monitoring smartwatches and wearables [59]; in addition, implantable pacemakers rely on this technology for seamless charging without surgery [3]. NFC devices, which can be used to trigger intervention messaging in particular spaces and environments on mobile devices, rely upon the sending and receipt of radio waves [60]. As these technologies continue to proliferate to support mHealth approaches, the likelihood of such devices fueling health concerns, negative expectations, and EHS symptoms via the nocebo effect is heightened. More generally, assuming that mHealth technologies and therapies are developed that are, in fact, safe and effective, these worries represent a critical implementational barrier to the dissemination and impact of mHealth. Thus, research on these topics may also be helpful to understand and address public distrust, thereby facilitating the expansion of safe and effective mHealth technologies (and providing the opportunity for such technologies to reach their potential for improving health).

## Psychological/Behavioral Concerns With Mobile Health Implementation

Roger’s [61] *Diffusion of Innovations Model* of the 1960s (among others) aimed to characterize individuals across a spectrum of psychological and behavioral dispositions toward adopting new technologies, from *early adopter* to *laggard*. However, given recent surges in the accessibility of mobile technologies, and that over 90% of global adults now own a mobile device [62], the many *mobile laggards* of recent decades have become potential mHealth consumers/users. The majority of people worldwide are surrounded by new technological systems and infrastructure (eg, video surveillance, open Wi-Fi, and long-term evolution networking) [5]. Separate from contributing to EHS and nocebo effects, the global push to *go mobile* has raised significant public concerns about data privacy and trust issues.

One-fourth of the world population logs on to Facebook at least once per month [63], and almost 88% of the global smartphone marketplace belongs to Google [64]. In recent years, such companies (among many others, such as Equifax [65] and Amazon [66]) have faced public scrutiny over their mass procurement, guarding, and utilization of personal and mobile usage data [67]. Much of these data are collected *passively*, as in, users are unaware of when their personal mobile device, browsing, or provided data are being collected, or how they are being harnessed (or used) by the associated company [68]. Many consumers, especially in high-income nations, view the procurement of ongoing, passively obtained personal, financial, and health information to be intrusive and an invasion of privacy [68,69].

Many tech giants (eg, Facebook and Google) assert that users fully accept their companies’ data privacy standards when they agree to the *terms and conditions* of signing up for a service [70]. Although such affirmation has in some cases been seen as legally binding [71], such data privacy considerations have also been widely criticized. mHealth technologies, particularly those that involve personal health information (PHI), may require greater attention to these data privacy concerns; this may result in better data management practices, but this also represents a potential barrier to implementation and adoption of mHealth technologies [72]. For example, clinically verified monitors for tracking ambulatory physiology (eg, blood pressure) face stringent US Food and Drug Administration standards for the transfer and storage of data to protect patient anonymity [73], and similar standards exist in other high-income nations [74]. Many private companies that manufacture medical-grade mHealth devices have also made their data privacy actions transparent on the Web, many aspiring to use layperson language [75-77]. Physicians have also requested clinician-focused educational materials about the security of mHealth technology’s data transfer and privacy, and easily delivered resources for ensuring enhanced transparency (to patients) about the safety of implementing new technologies into care [6]. Yet given the public distrust fueled by concerns with the tech giants’ usage of individuals’ mobile data, trust in upcoming mHealth technologies may dwindle—if so, such worries and concerns may slow the adoption and uptake of such devices. Indeed, researchers have noted considerable variability in perceived safety and security of mHealth apps and similar products [31].

## Mobile Health Innovations May Fuel Avoidance Behaviors

Avoidance behaviors are characterized by protective, sometimes proactive, actions toward avoiding situations that may be perceived as harmful to well-being [78]. In terms of innovative technologies, Technology Threat Avoidance Theory describes consumers’ actions for avoiding technological entities that are perceived to cause harmful outcomes, especially individual harms related to information and data security [32,79]. Although many mHealth technologies have secure collection and responsible usage of patient data for clinical purposes [7], broader concerns regarding general consumer security (eg, of big data procurement) are likely to persist even as mHealth continues to be more integrated into clinical prevention and intervention approaches—perhaps resulting in increased avoidance behaviors.

Worries regarding *data insecurity* [67] are certainly not constrained to mHealth. For example, SmartHome technologies (ie, wireless monitoring of heating and air conditioning, locks, and surveillance systems in homes, among other capabilities) are increasingly prevalent in high-income nations and require the transfer of intimate home-based environmental data and video streams between mobile devices [80]. In addition, social media utilization increased by 13% globally from 2017 to 2018, with most platforms being accessed via mobile devices [81], and the background monitoring of smartphone usage statistics and *digital footprints* is at an all-time high [82]. Multiple private and public technology companies, including those invested in mHealth and other consumer innovations, will benefit from a more nuanced and evidence-based understanding and accurate evaluation of perceived data security (or lack thereof) and its impact on avoidance behaviors.

## Societal Concerns With Mobile Health Implementation

For mHealth technologies to reach their full potential on a global level, they must be trusted, accepted, and adopted by society at large. One implementation factor positively associated with the adoption of newly innovative technologies to society is overall consumer *acceptability*. Multiple well-regarded models have been established to explicate the relationship between consumer acceptability and adoption of new technologies, including the Technology Acceptance Model [83] and the Consumer Acceptance of Technology model [84]. Acceptability is purported to be a multifaceted concept, with agreeability, satisfaction, and willingness to engage with a device being common constructs associated with measuring how *acceptable* a product is to an end user [19]. It can also be measured at different timepoints, including prior to use, to predict adherence with a new technology (eg, *hypothetical*, *a priori*, or *prospective* acceptability) and after (eg, *actual*, *experienced*, or *retrospective* acceptability) the technology has been introduced to the user [85,86]. Higher levels of consumer acceptability (both before and after engaging with a device) are associated with higher sustained levels of uptake to using technological systems [83,84].

The concern here is that for mHealth, we have strong reasons to believe that some significant fraction of potential consumers may have worries regarding potential negative health effects and data security and privacy, particularly as they see (likely nonrepresentative, but very powerful) media stories consistent with their concerns. In such cases, it appears likely that this will limit consumers’ acceptance of innovative mHealth approaches, their willingness to engage with mHealth, and their satisfaction with the technology (ie, devices thought to elicit negative health effects or foster data privacy concerns will not likely be satisfactory to the average consumer). Taken together, these effects will limit consumer mHealth acceptability, which, in turn, limits social uptake of the technology overall. If the individuals who can benefit from mHealth do not accept the technology, particularly when based on unfounded concerns, this may impede the larger societal acceptance and capacity of mHealth to reach its potential for public good as a tool for enhancing human health.

## Issues for Taking Mobile Health to Market

Government agencies tasked with ensuring public safety and health are likely to pay attention to any data privacy concerns or perceived negative health effects of mHealth products, even in the absence of a strong scientific base to support such worries. Addressing these concerns can be costly. For example, investigating complaints about wind farm–related EHS is anticipated to cost the Australian government more than Aus $2 million (US $1.2 million dollars) [23]. Chapman and Crichton [23] note that unfounded public concerns not only cause major delays in the rollout of beneficial technology but also impede the pace of global technological advancements. A growing rollout of mHealth to market in high-income nations in the near future may face similar scrutiny, potentially requiring taxpayer dollars, reducing government interest in supporting such technologies, and ultimately slowing the development and implementation of mHealth.

In addition, reported concerns with mHealth technologies can result in legal ramifications for product developers. For example, in the case of EHS, some sufferers have filed lawsuits against producers and local governing bodies in an attempt to rid their environments of various types of devices that emit electromagnetic radiation [43]. As similar public perceptions could evolve around mHealth, manufacturers who would otherwise be well fit to mass produce such technologies may be hesitant to embark on such an endeavor for fear of litigation, negative publicity, and lawsuits. Alternatively, if such concerns do emerge about a recently marketed product, the resultant legal and financial challenges may be beyond the capacity of the business to manage. In addition, extending prior discussion on this point, in recent years, tech giants such as Facebook and Apple have faced class-action legal suits regarding data privacy issues, in turn, contributing to publicized distrust and worry with associated apps and products [68,87].

As a function of reduced acceptability and uptake, the mHealth product market may be limited or may fail to show the expected growth. As has happened in the past when unfounded privacy and health concerns have been disseminated to the public, otherwise safe products are seen as unacceptable and these new technologies are avoided by consumers [23,83,84]. This, in turn, limits sales, meaning producers suffer low return on research and development expenditures. Subsequently, product developers halt manufacturing, shifting their resources and interests toward more acceptable products. The compounded scenario of distrust in mobile technologies, low acceptability limiting social capital and market reach, and hence, low producer investment in developing such devices, all reduce the likelihood of mHealth reaching its full potential for enhancing public health.

## Social and Behavioral Science Solutions to Address Mobile Health Implementation

To better understand and recalibrate public concerns surrounding emerging mHealth technologies, we propose a biopsychosocial framework derived from previous work to investigate these human-centered implementation factors. Such research can inform proactive prevention efforts, aimed at reducing the likelihood of negative expectations, nocebo effects, data and privacy concerns, and overall social worries, regulations, and barriers to taking mHealth products to the market.

Initially, we propose that public perceptions surrounding the safety and security of mHealth technologies be systematically evaluated. Specifically, such assessments should evaluate the aforementioned biopsychosocial constructs, and recruitment should be targeted at the population of interest for mHealth implementation. For example, populations with widespread uptake of mHealth technologies (eg, high-income nations like the United States [87]) may be of value for exploring individual and societal health concerns as they relate to innovative mHealth products. Such findings may translate to informative precedent for assessing such research questions in additional developing country populations where significant funding and infrastructure improvements have been recently proposed for mHealth expansion [88].

Next, after evaluating the target populations’ concerns with new mHealth technologies, we propose identifying any concerns that appear unfounded or overstated and developing evidence-based communication strategies that aim to minimize these misperceptions regarding biophysiological and psychological/behavioral risks related to mHealth implementation. In turn, these communications should enhance acceptability and self-efficacy for utilization of such technologies. Our view is that for meaningful impact, such interventions should focus on addressing the target population’s most prevalent technological misperceptions and should be developed using theory-based procedures. The Health Belief Model [89], the Theory of Planned Behavior [90], and the Communication Persuasion Model [91] may be helpful evidence-based theoretical approaches to employ in this context.

Finally, we assert that evidence-informed dissemination efforts of these theory-based communications (to the population of interest) are helpful to achieve the population-level effects for *preventing* additional future mHealth concerns. Providing consumers with accurate information, knowledge, and efficacy affirmations that appropriately (ie, evidence-based) allay potential worries, enhance trust, and facilitate the overall uptake of beneficial mHealth technologies are needed for this technology reaching its potential to improve health. Such public outreach and intervention endeavors will require determining the most appropriate and wide-reaching dissemination platform for the population of interest (eg, social media vs physician communication), the optimal frequency of communications, and how to design and tailor the specific content in such communications.

## Conclusions

New technologies can afford opportunities for improving societal function and individual well-being. mHealth technology holds the potential to have a large positive global impact on health. However, scientifically unsupported concerns surrounding electromagnetic radiation exposure may produce negative health outcomes via nocebo effects; coupled with data privacy issues and larger social implications, the array of related worries may hinder the development and implementation of mHealth technologies. We argue it is thus essential to carefully attend to these potential concerns, including distinguishing between unfounded mHealth-related worries and risks from evidence-based concerns and then evaluate and address the unfounded mHealth concerns. Some apprehension about new digital platforms (eg, wireless-powered pacemakers) may be expected, especially when the technology is very new and not widely documented or disseminated; it may also be the case that concerns, and avoidance may even serve to be proactively protective in some situations (eg, data security of PHI). Notwithstanding that some worries are entirely appropriate and adaptive, our goal here is to outline areas where unwarranted negative expectations and public concerns regarding mHealth are more likely. We hope that such attention may result in increased uptake of safe and effective mHealth, facilitating the reach and positive impact of these exciting new technologies.

This work was supported in part by the National Science Foundation Nanoscience Engineering Research Center for Advanced Self-Powered Systems of Integrated Sensors and Technologies (EEC-1160483).

## Abbreviations

EHS: electromagnetic hypersensitivity syndrome
EMF: electromagnetic field
mHealth: mobile health
NFC: near-field communication
PHI: personal health information

## References

[1] Kumar S, Nilsen WJ, Abernethy A, Atienza A, Patrick K, Pavel M, Riley WT, Shar A, Spring B, Spruijt-Metz D, Hedeker D, Honavar V, Kravitz R, Lefebvre RC, Mohr DC, Murphy SA, Quinn C, Shusterman V, Swendeman D (2013). Mobile health technology evaluation: the mHealth evidence workshop. Am J Prev Med.

[2] Burke LE, Ma J, Azar KM, Bennett GG, Peterson ED, Zheng Y, Riley W, Stephens J, Shah S, Suffoletto B, Turan TN, Spring B, Steinberger J, Quinn C, American Heart Association Publications Committee of the Council on Epidemiology and Prevention‚ Behavior Change Committee of the Council on Cardiometabolic Health‚ Council on Cardiovascular and Stroke Nursing‚ Council on Functional Genomics and Translational Biology‚ Council on Quality of Care and Outcomes Research‚Stroke Council (2015). Current science on consumer use of mobile health for cardiovascular disease prevention: a scientific statement from the American Heart Association. Circulation.

[3] Andreu-Perez J, Leff DR, Ip HM, Yang G (2015). From wearable sensors to smart implants--toward pervasive and personalized healthcare. IEEE Trans Biomed Eng.

[4] McCallum C, Rooksby J, Gray CM (2018). Evaluating the impact of physical activity apps and wearables: interdisciplinary review. JMIR Mhealth Uhealth.

[5] Adibi S, Mobasher A, Tofigh T (2014). LTE networking: extending the reach for sensors in mHealth applications. Trans Emerg Telecommun Technol.

[6] Ventola CL (2014). Mobile devices and apps for health care professionals: uses and benefits. Pharm Ther.

[7] Baig MM, GholamHosseini H, Connolly MJ (2015). Mobile healthcare applications: system design review, critical issues and challenges. Australas Phys Eng Sci Med.

[8] Charani E, Gharbi M, Moore L, Castro-Sanchéz E, Lawson W, Gilchrist M, Holmes AH (2017). Effect of adding a mobile health intervention to a multimodal antimicrobial stewardship programme across three teaching hospitals: an interrupted time series study. J Antimicrob Chemother.

[9] Krohn R (2015). mHealth: a pathway to the intelligent hospital. Mhealth.

[10] Lin JC (2013). Wireless power transfer for mobile applications, and health effects [Telecommunications Health and Safety]. IEEE Antennas Propag Mag.

[11] Ghorai K, Akter S, Khatun F, Ray P (2014). mHealth for smoking cessation programs: a systematic review. J Pers Med.

[12] Kemp CG, Velloza J (2018). Implementation of eHealth interventions across the HIV care cascade: a review of recent research. Curr HIV/AIDS Rep.

[13] Vervloet M, Linn AJ, van Weert JC, de Bakker DH, Bouvy ML, van Dijk L (2012). The effectiveness of interventions using electronic reminders to improve adherence to chronic medication: a systematic review of the literature. J Am Med Inform Assoc.

[14] Peiris D, Praveen D, Johnson C, Mogulluru K (2014). Use of mHealth systems and tools for non-communicable diseases in low- and middle-income countries: a systematic review. J Cardiovasc Transl Res.

[15] Hull TD, Mahan K (2017). A study of asynchronous mobile-enabled SMS text psychotherapy. Telemed J E Health.

[16] Bauer MS, Damschroder L, Hagedorn H, Smith J, Kilbourne AM (2015). An introduction to implementation science for the non-specialist. BMC Psychol.

[17] Mummah SA, Robinson TN, King AC, Gardner CD, Sutton S (2016). IDEAS (integrate, design, assess, and share): a framework and toolkit of strategies for the development of more effective digital interventions to change health behavior. J Med Internet Res.

[18] Schnall R, Rojas M, Bakken S, Brown W, Carballo-Dieguez A, Carry M, Gelaude D, Mosley JP, Travers J (2016). A user-centered model for designing consumer mobile health (mHealth) applications (apps). J Biomed Inform.

[19] Materia F, Downs DS, Smyth J (2019). Conceptualizing Acceptability in mHealth Research. Proceedings of the 6th Biannual Meeting of the Society for Ambulatory Assessment.

[20] Brent RL (1980). X-ray, microwave, and ultrasound: the real and unreal hazards. Pediatr Ann.

[21] Bren S (1996). Historical introduction to EMF health effects. IEEE Eng Med Biol Mag.

[22] Oyewopo AO, Olaniyi SK, Oyewopo CI, Jimoh AT (2017). Radiofrequency electromagnetic radiation from cell phone causes defective testicular function in male Wistar rats. Andrologia.

[23] Chapman S, Crichton F (2017). Wind Turbine Syndrome: A Communicated Disease. First Edition.

[24] (2014). The Daily Mail.

[25] Foster KR (2019). Scientific American Blogs.

[26] Moskowitz JM (2019). Scientific American Blogs.

[27] Di Ciaula A (2018). Towards 5G communication systems: are there health implications?. Int J Hyg Environ Health.

[28] Faasse K, Petrie KJ (2013). The nocebo effect: patient expectations and medication side effects. Postgrad Med J.

[29] Petrie K, Wessely S (2002). Modern worries, new technology, and medicine. Br Med J.

[30] Schmidt DC (2018). Digital Content Next (DCN).

[31] Boulos MN, Brewer AC, Karimkhani C, Buller DB, Dellavalle RP (2014). Mobile medical and health apps: state of the art, concerns, regulatory control and certification. Online J Public Health Inform.

[32] Liang H, Xue Y (2009). Avoidance of information technology threats: a theoretical perspective. Manag Inf Syst Q.

[33] King DK, Glasgow RE, Leeman-Castillo B (2010). Reaiming RE-AIM: using the model to plan, implement, and evaluate the effects of environmental change approaches to enhancing population health. Am J Public Health.

[34] Damschroder LJ, Aron DC, Keith RE, Kirsh SR, Alexander JA, Lowery JC (2009). Fostering implementation of health services research findings into practice: a consolidated framework for advancing implementation science. Implement Sci.

[35] Rubin GJ, Munshi JD, Wessely S (2005). Electromagnetic hypersensitivity: a systematic review of provocation studies. Psychosom Med.

[36] Filipkowski KB, Smyth JM, Rutchick AM, Santuzzi AM, Adya M, Petrie KJ, Kaptein AA (2010). Do healthy people worry? Modern health worries, subjective health complaints, perceived health, and health care utilization. Int J Behav Med.

[37] Bennett P, Calman K, Curtis S, Fischbacher-Smith D, Bennett P, Calman K, Curtis S, Fischbacher-Smith D (2010). Understanding public responses to risk: issues around policy and practice. Risk Communication and Public Health.

[38] Schreier N, Huss A, Röösli M (2006). The prevalence of symptoms attributed to electromagnetic field exposure: a cross-sectional representative survey in Switzerland. Soz Praventivmed.

[39] (2019). Australian Radiation Protection and Nuclear Safety Agency.

[40] Hussain A, Ali S, Ahmed M, Hussain S (2018). The anti-vaccination movement: a regression in modern medicine. Cureus.

[41] Ruden D, Rasouli P, Lu X (2007). Potential long-term consequences of fad diets on health, cancer, and longevity: lessons learned from model organism studies. Technol Cancer Res Treat.

[42] Eldridge-Thomas B, Rubin GJ (2013). Idiopathic environmental intolerance attributed to electromagnetic fields: a content analysis of British newspaper reports. PLoS One.

[43] Hall H (2015). Science-Based Medicine.

[44] Gruber MJ, Palmquist E, Nordin S (2018). Characteristics of perceived electromagnetic hypersensitivity in the general population. Scand J Psychol.

[45] Kwon M, Kim S, Koo J, Choi J, Kim D (2012). EHS Subjects Do Not Perceive RF EMF Emitted From Smart Phones Better Than Non-EHS Subjects. Proceedings of the 2012 Annual International Conference of the IEEE Engineering in Medicine and Biology Society.

[46] Kwon MK, Choi JY, Kim SK, Yoo TK, Kim DW (2012). Effects of radiation emitted by WCDMA mobile phones on electromagnetic hypersensitive subjects. Environ Health.

[47] Kwon MS, Koivisto M, Laine M, Hämäläinen H (2008). Perception of the electromagnetic field emitted by a mobile phone. Bioelectromagnetics.

[48] Röösli M (2008). Radiofrequency electromagnetic field exposure and non-specific symptoms of ill health: a systematic review. Environ Res.

[49] Röösli M, Hug K (2011). Wireless communication fields and non-specific symptoms of ill health: a literature review. Wien Med Wochenschr.

[50] Rubin GJ, Hillert L, Nieto-Hernandez R, van Rongen E, Oftedal G (2011). Do people with idiopathic environmental intolerance attributed to electromagnetic fields display physiological effects when exposed to electromagnetic fields? A systematic review of provocation studies. Bioelectromagnetics.

[51] Eltiti S, Wallace D, Russo R, Fox E (2015). Aggregated data from two double-blind base station provocation studies comparing individuals with idiopathic environmental intolerance with attribution to electromagnetic fields and controls. Bioelectromagnetics.

[52] Barsky AJ, Saintfort R, Rogers MP, Borus JF (2002). Nonspecific medication side effects and the nocebo phenomenon. J Am Med Assoc.

[53] Faasse K, Helfer SG, Barnes K, Colagiuri B, Geers AL (2019). Experimental assessment of nocebo effects and nocebo side effects: definitions, study design, and implications for psychiatry and beyond. Front Psychiatry.

[54] Faasse K, Huynh A, Pearson S, Geers A, Helfer S, Colagiuri B (2019). The influence of side effect information framing on nocebo effects. Ann Behav Med.

[55] Mahr A, Golmard C, Pham E, Iordache L, Deville L, Faure P (2017). Types, frequencies, and burden of nonspecific adverse events of drugs: analysis of randomized placebo-controlled clinical trials. Pharmacoepidemiol Drug Saf.

[56] Reeves RR, Ladner ME, Hart RH, Burke RS (2007). Nocebo effects with antidepressant clinical drug trial placebos. Gen Hosp Psychiatry.

[57] Häuser W, Hansen E, Enck P (2012). Nocebo phenomena in medicine: their relevance in everyday clinical practice. Dtsch Arztebl Int.

[58] Petrie KJ, Faasse K, Crichton F, Grey A (2014). How common are symptoms? Evidence from a New Zealand national telephone survey. BMJ Open.

[59] Miamura K, Miyaji Y, Ohmura R (2017). Feasibility Study on Wireless Power Transfer for Wearable Devices. Proceedings of the 2017 ACM International Symposium on Wearable Computers.

[60] Strömmer E, Kaartinen J, Pärkkä J, Ylisaukko-Oja A, Korhonen I (2006). Application of near field communication for health monitoring in daily life. Conf Proc IEEE Eng Med Biol Soc.

[61] Dearing JW (2009). Applying diffusion of innovation theory to intervention development. Res Soc Work Pract.

[62] (2018). Pew Research Center.

[63] Popper B (2017). The Verge.

[64] Kharpal A (2016). CNBC.

[65] Zou Y, Mhaidli AH, Mccall A, Schaub F (2018). 'I’ve Got Nothing to Lose': Consumers’ Risk Perceptions and Protective Actions after the Equifax Data Breach. Proceedings of the Fourteenth Symposium on Usable Privacy and Security.

[66] Bhattarai A (2018). The Washington Post.

[67] Rand-Hendriksen M (2015). LinkedIn.

[68] Andreou A, Silva M, Benevenuto F, Goga O, Loiseau P, Mislove A (2019). Measuring the Facebook Advertising Ecosystem. Proceedings of the 2019 Network and Distributed Systems Security Symposium.

[69] Harwell D (2018). The Washington Post.

[70] Steinfeld N (2016). 'I agree to the terms and conditions': (How) do users read privacy policies online? An eye-tracking experiment. Comput Human Behav.

[71] Budnitz ME (2000). The Reading Room.

[72] Gomes R, Lapão LV (2008). The adoption of IT security standards in a healthcare environment. Stud Health Technol Inform.

[73] (2017). US Food and Drug Administration.

[74] Medicines & Health Products Regulatory Agency (2018). The Government of UK.

[75] (2019). Alivecor.

[76] (2019). Polar.

[77] (2017). Spire Healthcare.

[78] Kazdin AE (1974). Covert modeling, model similarity, and reduction of avoidance behavior. Behav Ther.

[79] Chen D, Zhao H (2012). Data Security and Privacy Protection Issues in Cloud Computing. Proceedings of the 2012 International Conference on Computer Science and Electronics Engineering.

[80] Chakravorty A, Wlodarczyk T, Rong C (2013). Privacy Preserving Data Analytics for Smart Homes. Proceedings of the 2013 IEEE Security and Privacy Workshops.

[81] Chaffey D (2019). Smart Insights.

[82] Ng V, Kent C (2018). The Conversation.

[83] Davis FD (1989). Perceived usefulness, perceived ease of use, and user acceptance of information technology. Manag Inf Syst Q.

[84] Kulviwat S, Bruner II GC, Kumar A, Nasco SA, Clark T (2007). Toward a unified theory of consumer acceptance technology. Psychol Mark.

[85] Berry N, Lobban F, Emsley R, Bucci S (2016). Acceptability of interventions delivered online and through mobile phones for people who experience severe mental health problems: A systematic review. J Med Internet Res.

[86] Sekhon M, Cartwright M, Francis JJ (2017). Acceptability of healthcare interventions: an overview of reviews and development of a theoretical framework. BMC Health Serv Res.

[87] Bassett DR, Freedson PS, John D (2019). Wearable activity trackers in clinical research and practice. Kinesiol Rev.

[88] Pillay Y, Motsoaledi PA (2018). Digital health in South Africa: innovating to improve health. BMJ Glob Health.

[89] Ahadzadeh AS, Sharif SP, Ong FS, Khong KW (2015). Integrating health belief model and technology acceptance model: an investigation of health-related internet use. J Med Internet Res.

[90] Ajzen I (2019). Web Hosting at UMass Amherst.

[91] McGuire WJ (1984). Public communication as a strategy for inducing health-promoting behavioral change. Prev Med.

